# Dimethano­lbis[*N*′-(3-pyridylmethyl­ene)benzohydrazide]sodium(I) iodide

**DOI:** 10.1107/S1600536808040750

**Published:** 2008-12-10

**Authors:** Lian-Sheng Cui, Han-Dong Yin, Ming-Lei Yang, Da-Qi Wang

**Affiliations:** a College of Chemistry and Chemical Engineering, Liaocheng University, Shandong 252059, People’s Republic of China

## Abstract

The molecule of the title compound, [Na(C_13_H_11_N_3_O)_2_(CH_3_OH)_2_]I, is non-planar, with the Na atom chelated by the O atoms and the N atoms of two *N*′-(3-pyridylmethyl­ene)benzohydrazide ligands and both O atoms of two methanol ligands. The asymmetric unit consists of one half-mol­ecule. The Na atom is located on a crystallographic centre of inversion. The six-coordinate Na atom adopts a distorted octa­hedral coordination. In the crystal structure, inter­molecular N—H⋯I and O—H⋯N hydrogen bonds link the mol­ecules into a two-dimensional network.

## Related literature

For general background, see: Lindoy *et al.* (1976 ▶). For bond-length data, see: Allen *et al.* (1987 ▶).
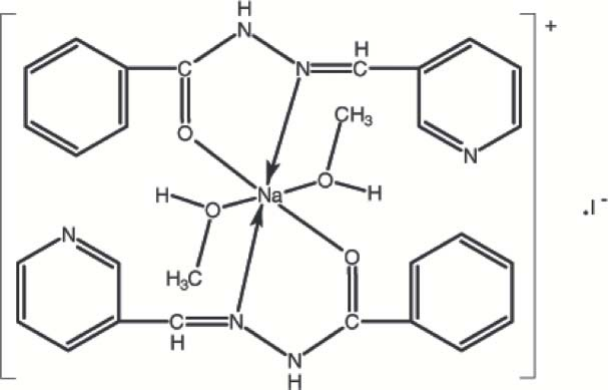


## Experimental

### 

#### Crystal data

[Na(C_13_H_11_N_3_O)_2_(CH_4_O)_2_]I
*M* *_r_* = 664.47
Monoclinic, 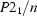

*a* = 8.6078 (15) Å
*b* = 13.1842 (16) Å
*c* = 13.2508 (17) Å
β = 101.6540 (10)°
*V* = 1472.8 (4) Å^3^
*Z* = 2
Mo *K*α radiation
μ = 1.15 mm^−1^
*T* = 298 (2) K
0.54 × 0.43 × 0.40 mm

#### Data collection

Bruker SMART CCD area detector diffractometer
Absorption correction: multi-scan (*SADABS*; Sheldrick, 1996 ▶) *T*_min_ = 0.577, *T*_max_ = 0.657
7140 measured reflections
2586 independent reflections
1919 reflections with *I* > 2σ(*I*)
*R*_int_ = 0.078

#### Refinement

*R*[*F* ^2^ > 2σ(*F* ^2^)] = 0.037
*wR*(*F* ^2^) = 0.104
*S* = 1.05
2586 reflections
185 parameters
H-atom parameters constrained
Δρ_max_ = 0.69 e Å^−3^
Δρ_min_ = −0.87 e Å^−3^

### 

Data collection: *SMART* (Siemens, 1996 ▶); cell refinement: *SAINT* (Siemens, 1996 ▶); data reduction: *SAINT*; program(s) used to solve structure: *SHELXS97* (Sheldrick, 2008 ▶); program(s) used to refine structure: *SHELXL97* (Sheldrick, 2008 ▶); molecular graphics: *SHELXTL* (Sheldrick, 2008 ▶); software used to prepare material for publication: *SHELXTL*.

## Supplementary Material

Crystal structure: contains datablocks I, global. DOI: 10.1107/S1600536808040750/at2678sup1.cif

Structure factors: contains datablocks I. DOI: 10.1107/S1600536808040750/at2678Isup2.hkl

Additional supplementary materials:  crystallographic information; 3D view; checkCIF report

## Figures and Tables

**Table:** 

Na1—O1	2.294 (3)
Na1—O2	2.344 (2)
Na1—N2	2.642 (3)

**Table:** 

O1—Na1—O2	86.74 (10)
O1—Na1—N2	65.34 (9)
O2—Na1—N2	88.40 (9)

**Table 2:** Hydrogen-bond geometry (Å, °)

*D*—H⋯*A*	*D*—H	H⋯*A*	*D*⋯*A*	*D*—H⋯*A*
N1—H1⋯I1^i^	0.86	3.03	3.816 (3)	153
O2—H2⋯N3^ii^	0.82	1.98	2.792 (5)	171

Symmetry codes: (i) 
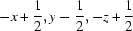
; (ii) 
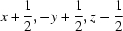
.

**Fig. 1.**
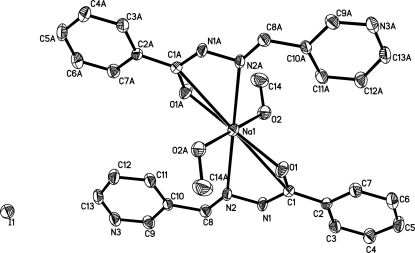
The structure of the title compound, showing 30% probability displacement ellipsoids and the atom-numbering scheme.

**Fig. 2.**
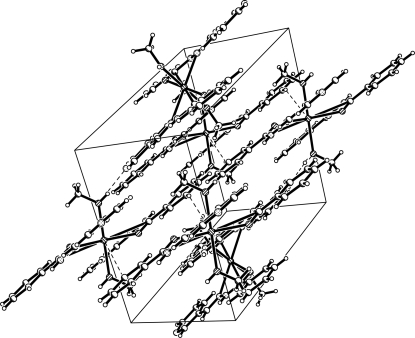
The crystal packing of the title compound, viewed approximately along the c axis.

## supplementary crystallographic information

### Comment

Schiff bases have been known as effective ligands for metal ions in the preparation of dyes for many years, liquid crystals and powerful corrosion inhibitors. Furthermore, they are used in the mechanism of many biochemical processes (Lindoy *et al.*, 1976). We report here the synthesis and crystal structure of the title compound (I).

The molecular structure of (I) is shown in Fig.1. The values of the geometric parameters in (I) are normal (Allen *et al.*, 1987) (Table 1). In the crystal structure, there exist two intermolecular N—H···I and O—H···N hydrogen bonds (Table 2). As seen in Fig.2, the molecules are linked into two-dimensional network.

### Experimental

A mixture of *N*'-(3-pyridylmethylene)benzohydrazide (3 mmol) and sodium methoxide (3 mmol) and bismuth iodide(1 mmol) in absolute ethanol (15 ml) was heated under reflux with stirring for 5 h and then filtered.The resulting clear colourless solution was diffused diethyl ether vapor at room temperature for 16 days, after which large colourless block-shaped crystals of the title complex suitable for X-ray diffraction analysis were obtained.

### Refinement

All H-atoms were positioned geometrically and refined using a riding model, with C—H = 0.96 Å (methylene) or 0.93 Å (aromatic),0.82 Å (hydroxyl) and *U*_iso_(H) =1.2*U*_eq_(C).

### Crystal data

**Table:**

[Na(C_13_H_11_N_3_O)_2_(CH_4_O)_2_]I	*F*(000) = 672.0
*M**_r_* = 664.47	*D*_x_ = 1.494 Mg m^−^^3^
Monoclinic, *P*2_1_/*n*	Mo *K*α radiation, λ = 0.71073 Å
Hall symbol: -P 2yn	Cell parameters from 3137 reflections
*a* = 8.6078 (15) Å	θ = 2.2–27.2°
*b* = 13.1842 (16) Å	µ = 1.15 mm^−^^1^
*c* = 13.2508 (17) Å	*T* = 298 K
β = 101.654 (1)°	Block, colourless
*V* = 1472.8 (4) Å^3^	0.54 × 0.43 × 0.40 mm
*Z* = 2	

### Data collection

**Table:**

Bruker SMART CCD area detector diffractometer	2586 independent reflections
Radiation source: fine-focus sealed tube	1919 reflections with *I* > 2σ(*I*)
graphite	*R*_int_ = 0.078
phi and ω scans	θ_max_ = 25.0°, θ_min_ = 2.2°
Absorption correction: multi-scan (*SADABS*; Sheldrick, 1996)	*h* = −10→9
*T*_min_ = 0.577, *T*_max_ = 0.657	*k* = −15→12
7140 measured reflections	*l* = −15→15

### Refinement

**Table:**

Refinement on *F*^2^	Secondary atom site location: difference Fourier map
Least-squares matrix: full	Hydrogen site location: inferred from neighbouring sites
*R*[*F*^2^ > 2σ(*F*^2^)] = 0.037	H-atom parameters constrained
*wR*(*F*^2^) = 0.104	*w* = 1/[σ^2^(*F*_o_^2^) + (0.028*P*)^2^ + 0.7317*P*] where *P* = (*F*_o_^2^ + 2*F*_c_^2^)/3
*S* = 1.05	(Δ/σ)_max_ < 0.001
2586 reflections	Δρ_max_ = 0.69 e Å^−^^3^
185 parameters	Δρ_min_ = −0.87 e Å^−^^3^
0 restraints	Extinction correction: *SHELXL*, Fc^*^=kFc[1+0.001xFc^2^λ^3^/sin(2θ)]^-1/4^
Primary atom site location: structure-invariant direct methods	Extinction coefficient: 0.0258 (18)

### Special details

**Table:**

Geometry. All e.s.d.'s (except the e.s.d. in the dihedral angle between two l.s. planes) are estimated using the full covariance matrix. The cell e.s.d.'s are taken into account individually in the estimation of e.s.d.'s in distances, angles and torsion angles; correlations between e.s.d.'s in cell parameters are only used when they are defined by crystal symmetry. An approximate (isotropic) treatment of cell e.s.d.'s is used for estimating e.s.d.'s involving l.s. planes.
Refinement. Refinement of *F*^2^ against ALL reflections. The weighted *R*-factor *wR* and goodness of fit *S* are based on *F*^2^, conventional *R*-factors *R* are based on *F*, with *F* set to zero for negative *F*^2^. The threshold expression of *F*^2^ > σ(*F*^2^) is used only for calculating *R*-factors(gt) *etc*. and is not relevant to the choice of reflections for refinement. *R*-factors based on *F*^2^ are statistically about twice as large as those based on *F*, and *R*- factors based on ALL data will be even larger.

### Fractional atomic coordinates and isotropic or equivalent isotropic displacement parameters (Å^2^)

**Table:**

	*x*	*y*	*z*	*U*_iso_*/*U*_eq_	
Na1	0.5000	0.5000	0.0000	0.0383 (5)	
I1	0.0000	0.5000	0.5000	0.0439 (2)	
N1	0.6395 (4)	0.2735 (2)	0.0500 (2)	0.0384 (8)	
H1	0.6445	0.2085	0.0462	0.046*	
N2	0.5180 (3)	0.3195 (2)	0.0883 (2)	0.0347 (7)	
N3	0.0867 (4)	0.2404 (3)	0.2536 (3)	0.0470 (9)	
O1	0.7451 (3)	0.42504 (19)	0.0231 (2)	0.0480 (7)	
O2	0.4427 (3)	0.4186 (2)	−0.16052 (19)	0.0492 (7)	
H2	0.4879	0.3694	−0.1793	0.074*	
C1	0.7503 (4)	0.3326 (3)	0.0187 (3)	0.0336 (8)	
C2	0.8797 (4)	0.2795 (3)	−0.0193 (3)	0.0319 (8)	
C3	0.8939 (5)	0.1758 (3)	−0.0264 (3)	0.0455 (10)	
H3	0.8179	0.1337	−0.0075	0.055*	
C4	1.0197 (6)	0.1342 (3)	−0.0613 (3)	0.0564 (12)	
H4	1.0281	0.0641	−0.0654	0.068*	
C5	1.1334 (5)	0.1946 (4)	−0.0902 (3)	0.0526 (11)	
H5	1.2187	0.1658	−0.1131	0.063*	
C6	1.1193 (5)	0.2980 (3)	−0.0848 (3)	0.0498 (11)	
H6	1.1948	0.3398	−0.1048	0.060*	
C7	0.9930 (4)	0.3401 (3)	−0.0497 (3)	0.0424 (9)	
H7	0.9842	0.4103	−0.0465	0.051*	
C8	0.4330 (5)	0.2585 (3)	0.1286 (3)	0.0382 (9)	
H8	0.4564	0.1895	0.1299	0.046*	
C9	0.2103 (5)	0.2198 (3)	0.2102 (3)	0.0434 (10)	
H9	0.2379	0.1522	0.2045	0.052*	
C10	0.3003 (4)	0.2933 (3)	0.1730 (3)	0.0329 (8)	
C11	0.2568 (5)	0.3933 (3)	0.1804 (3)	0.0439 (10)	
H11	0.3129	0.4450	0.1561	0.053*	
C12	0.1293 (5)	0.4150 (3)	0.2242 (3)	0.0487 (10)	
H12	0.0978	0.4819	0.2299	0.058*	
C13	0.0492 (5)	0.3378 (3)	0.2592 (3)	0.0471 (10)	
H13	−0.0368	0.3542	0.2887	0.057*	
C14	0.3307 (7)	0.4569 (4)	−0.2446 (3)	0.0740 (15)	
H14A	0.2857	0.5185	−0.2245	0.111*	
H14B	0.2481	0.4078	−0.2655	0.111*	
H14C	0.3821	0.4705	−0.3011	0.111*	

### Atomic displacement parameters (Å^2^)

**Table:**

	*U*^11^	*U*^22^	*U*^33^	*U*^12^	*U*^13^	*U*^23^
Na1	0.0414 (12)	0.0344 (12)	0.0424 (11)	0.0097 (9)	0.0161 (9)	−0.0003 (9)
I1	0.0536 (3)	0.0320 (3)	0.0480 (3)	0.00088 (16)	0.01502 (17)	−0.00036 (16)
N1	0.0429 (19)	0.0277 (16)	0.0514 (19)	0.0058 (14)	0.0258 (16)	−0.0028 (14)
N2	0.0336 (17)	0.0352 (17)	0.0395 (17)	0.0050 (14)	0.0178 (14)	−0.0020 (13)
N3	0.046 (2)	0.054 (2)	0.047 (2)	−0.0037 (17)	0.0229 (17)	0.0043 (16)
O1	0.0430 (16)	0.0313 (15)	0.0748 (19)	0.0031 (12)	0.0237 (14)	−0.0019 (13)
O2	0.0611 (19)	0.0450 (17)	0.0432 (15)	0.0100 (14)	0.0145 (14)	−0.0067 (13)
C1	0.033 (2)	0.033 (2)	0.0365 (19)	0.0057 (17)	0.0096 (16)	0.0026 (16)
C2	0.034 (2)	0.033 (2)	0.0294 (18)	0.0052 (17)	0.0082 (15)	−0.0014 (15)
C3	0.057 (3)	0.035 (2)	0.052 (2)	0.0053 (19)	0.028 (2)	0.0038 (18)
C4	0.067 (3)	0.044 (3)	0.067 (3)	0.018 (2)	0.035 (2)	0.002 (2)
C5	0.044 (3)	0.069 (3)	0.050 (3)	0.013 (2)	0.024 (2)	−0.006 (2)
C6	0.041 (2)	0.064 (3)	0.048 (2)	−0.006 (2)	0.020 (2)	−0.003 (2)
C7	0.042 (2)	0.040 (2)	0.047 (2)	−0.0017 (19)	0.0153 (18)	0.000 (2)
C8	0.044 (2)	0.032 (2)	0.042 (2)	0.0015 (17)	0.0164 (19)	0.0000 (16)
C9	0.053 (3)	0.037 (2)	0.044 (2)	−0.004 (2)	0.0186 (19)	0.0010 (19)
C10	0.031 (2)	0.039 (2)	0.0303 (18)	−0.0002 (17)	0.0116 (15)	0.0001 (16)
C11	0.054 (3)	0.037 (2)	0.048 (2)	−0.0022 (19)	0.027 (2)	−0.0001 (18)
C12	0.056 (3)	0.039 (2)	0.058 (3)	0.004 (2)	0.030 (2)	−0.005 (2)
C13	0.041 (2)	0.063 (3)	0.042 (2)	0.001 (2)	0.0216 (18)	−0.003 (2)
C14	0.095 (4)	0.076 (3)	0.049 (3)	0.012 (3)	0.009 (3)	0.007 (3)

### Geometric parameters (Å, °)

**Table:**

Na1—O1	2.294 (3)	C4—C5	1.375 (6)
Na1—O1^i^	2.294 (3)	C4—H4	0.9300
Na1—O2^i^	2.344 (2)	C5—C6	1.371 (6)
Na1—O2	2.344 (2)	C5—H5	0.9300
Na1—N2	2.642 (3)	C6—C7	1.382 (5)
Na1—N2^i^	2.642 (3)	C6—H6	0.9300
Na1—C1^i^	3.059 (4)	C7—H7	0.9300
N1—C1	1.360 (4)	C8—C10	1.461 (5)
N1—N2	1.390 (4)	C8—H8	0.9300
N1—H1	0.8600	C9—C10	1.391 (5)
N2—C8	1.276 (5)	C9—H9	0.9300
N3—C13	1.330 (5)	C10—C11	1.379 (5)
N3—C9	1.335 (5)	C11—C12	1.371 (5)
O1—C1	1.222 (4)	C11—H11	0.9300
O2—C14	1.412 (5)	C12—C13	1.362 (6)
O2—H2	0.8200	C12—H12	0.9300
C1—C2	1.487 (5)	C13—H13	0.9300
C2—C3	1.377 (5)	C14—H14A	0.9600
C2—C7	1.382 (5)	C14—H14B	0.9600
C3—C4	1.375 (6)	C14—H14C	0.9600
C3—H3	0.9300		
			
O1—Na1—O1^i^	180.0	C4—C3—H3	119.8
O1—Na1—O2^i^	93.26 (10)	C2—C3—H3	119.8
O1^i^—Na1—O2^i^	86.74 (10)	C3—C4—C5	121.1 (4)
O1—Na1—O2	86.74 (10)	C3—C4—H4	119.5
O1^i^—Na1—O2	93.26 (10)	C5—C4—H4	119.5
O2^i^—Na1—O2	180.0	C6—C5—C4	119.0 (4)
O1—Na1—N2	65.34 (9)	C6—C5—H5	120.5
O1^i^—Na1—N2	114.66 (9)	C4—C5—H5	120.5
O2^i^—Na1—N2	91.60 (9)	C5—C6—C7	120.0 (4)
O2—Na1—N2	88.40 (9)	C5—C6—H6	120.0
O1—Na1—N2^i^	114.66 (9)	C7—C6—H6	120.0
O1^i^—Na1—N2^i^	65.34 (9)	C2—C7—C6	121.0 (4)
O2^i^—Na1—N2^i^	88.40 (9)	C2—C7—H7	119.5
O2—Na1—N2^i^	91.60 (9)	C6—C7—H7	119.5
N2—Na1—N2^i^	180.00 (6)	N2—C8—C10	122.1 (3)
O1—Na1—C1^i^	159.30 (9)	N2—C8—H8	118.9
O1^i^—Na1—C1^i^	20.70 (9)	C10—C8—H8	118.9
O2^i^—Na1—C1^i^	76.04 (9)	N3—C9—C10	124.1 (4)
O2—Na1—C1^i^	103.96 (9)	N3—C9—H9	118.0
N2—Na1—C1^i^	131.53 (9)	C10—C9—H9	118.0
N2^i^—Na1—C1^i^	48.47 (9)	C11—C10—C9	117.5 (3)
C1—N1—N2	119.1 (3)	C11—C10—C8	125.1 (3)
C1—N1—H1	120.4	C9—C10—C8	117.4 (3)
N2—N1—H1	120.4	C12—C11—C10	118.8 (4)
C8—N2—N1	114.5 (3)	C12—C11—H11	120.6
C8—N2—Na1	140.1 (3)	C10—C11—H11	120.6
N1—N2—Na1	102.3 (2)	C13—C12—C11	119.4 (4)
C13—N3—C9	116.3 (3)	C13—C12—H12	120.3
C1—O1—Na1	117.7 (2)	C11—C12—H12	120.3
C14—O2—Na1	122.5 (3)	N3—C13—C12	123.9 (4)
C14—O2—H2	109.5	N3—C13—H13	118.0
Na1—O2—H2	127.7	C12—C13—H13	118.0
O1—C1—N1	121.5 (3)	O2—C14—H14A	109.5
O1—C1—C2	121.5 (3)	O2—C14—H14B	109.5
N1—C1—C2	117.0 (3)	H14A—C14—H14B	109.5
C3—C2—C7	118.4 (3)	O2—C14—H14C	109.5
C3—C2—C1	125.0 (3)	H14A—C14—H14C	109.5
C7—C2—C1	116.6 (3)	H14B—C14—H14C	109.5
C4—C3—C2	120.4 (4)		
			
C1—N1—N2—C8	−170.8 (3)	N2—N1—C1—C2	178.6 (3)
C1—N1—N2—Na1	25.1 (3)	O1—C1—C2—C3	179.9 (4)
O1—Na1—N2—C8	175.8 (4)	N1—C1—C2—C3	1.0 (5)
O1^i^—Na1—N2—C8	−4.2 (4)	O1—C1—C2—C7	−0.2 (5)
O2^i^—Na1—N2—C8	82.9 (4)	N1—C1—C2—C7	−179.1 (3)
O2—Na1—N2—C8	−97.1 (4)	C7—C2—C3—C4	1.1 (6)
C1^i^—Na1—N2—C8	10.2 (4)	C1—C2—C3—C4	−179.0 (4)
O1—Na1—N2—N1	−27.19 (19)	C2—C3—C4—C5	−0.3 (6)
O1^i^—Na1—N2—N1	152.81 (19)	C3—C4—C5—C6	−0.6 (7)
O2^i^—Na1—N2—N1	−120.0 (2)	C4—C5—C6—C7	0.6 (6)
O2—Na1—N2—N1	60.0 (2)	C3—C2—C7—C6	−1.1 (6)
C1^i^—Na1—N2—N1	167.28 (18)	C1—C2—C7—C6	179.1 (4)
O2^i^—Na1—O1—C1	122.2 (3)	C5—C6—C7—C2	0.2 (6)
O2—Na1—O1—C1	−57.8 (3)	N1—N2—C8—C10	179.9 (3)
N2—Na1—O1—C1	31.9 (3)	Na1—N2—C8—C10	−24.9 (6)
N2^i^—Na1—O1—C1	−148.1 (3)	C13—N3—C9—C10	0.9 (6)
C1^i^—Na1—O1—C1	180.0	N3—C9—C10—C11	−1.0 (6)
O1—Na1—O2—C14	−153.5 (3)	N3—C9—C10—C8	179.3 (4)
O1^i^—Na1—O2—C14	26.5 (3)	N2—C8—C10—C11	−2.9 (6)
N2—Na1—O2—C14	141.2 (3)	N2—C8—C10—C9	176.7 (4)
N2^i^—Na1—O2—C14	−38.8 (3)	C9—C10—C11—C12	0.5 (5)
C1^i^—Na1—O2—C14	8.6 (4)	C8—C10—C11—C12	−179.9 (4)
Na1—O1—C1—N1	−32.3 (4)	C10—C11—C12—C13	0.0 (6)
Na1—O1—C1—C2	148.9 (3)	C9—N3—C13—C12	−0.3 (6)
N2—N1—C1—O1	−0.3 (5)	C11—C12—C13—N3	−0.1 (7)

Symmetry codes: (i) −*x*+1, −*y*+1, −*z*.

### Hydrogen-bond geometry (Å, °)

**Table:**

*D*—H···*A*	*D*—H	H···*A*	*D*···*A*	*D*—H···*A*
N1—H1···I1^ii^	0.86	3.03	3.816 (3)	153
O2—H2···N3^iii^	0.82	1.98	2.792 (5)	171

Symmetry codes: (ii) −*x*+1/2, *y*−1/2, −*z*+1/2; (iii) *x*+1/2, −*y*+1/2, *z*−1/2.
